# The Reduction of Pathogen Load on Ross 708 Broilers when Using Different Sources of Commercial Peracetic Acid Sanitizers in a Pilot Processing Plant

**DOI:** 10.3390/microorganisms7110503

**Published:** 2019-10-29

**Authors:** Kristina M. Feye, Dana K. Dittoe, Zhaohao Shi, Jessica Woitte, Casey M. Owens, Mike H. Kogut, Steven C. Ricke

**Affiliations:** 1 Southern Plains Agricultural Research Center, United States Department of Agriculture-Agricultural Research Unit, College Station, TX 77845, USA; Feye@uark.edu (K.M.F.); mike.kogut@usda.gov (M.H.K.); 2 Center for Food Safety and Department of Poultry Science, University of Arkansas, Fayetteville, AR 72704, USA; DKDittoe@email.uark.edu (D.K.D.); hao.shi.368@gmail.com (Z.S.); jlwoitte@email.uark.edu (J.W.); 3 Department of Poultry Science, University of Arkansas, Fayetteville, AR 72704, USA; cmowens@uark.edu

**Keywords:** *Salmonella*, *Campylobacter*, poultry processing, PAA

## Abstract

Peracetic acid (PAA) in poultry processing is not necessarily the same from company to company. Anecdotal evidence suggests that PeraClean may be more stable compared to the competition; however, it is not known what impact potential differences in chemical stability may have. In order to evaluate the antimicrobial effects of PAA, one PAA (PeraClean, P) was qualitatively compared against two competitor products (Competitors 1 and 2, C1 and C2) at the University of Arkansas Pilot Processing Plant. A total of 150 Ross 708 broilers (42 d) were used in the current study. Briefly, prior to treatment, 10 birds were sampled post-evisceration (C). Then, one of four treatment groups per PAA were applied (A1, A2, B1, and B2). The birds were dipped in either 400 ppm or 600 ppm PAA (A or B), chilled in either 25 ppm or 45 ppm PAA (1 or 2), and then manually agitated in 400 mL of nBPW for 1 min. There were 10 birds per treatment group in total. The resulting rinsates were transported to the Center for Food Safety and assessed for total microbiological load with total aerobic plate counts (Trypticase Soy Agar; APC), coliforms, (Eosin Methylene Blue Media; EMB), *Salmonella* (Xylose Lysine Deoxycholate agar, XLD), and *Campylobacter* (modified Charcoal Cefoperazone Deoxycholate Agar, mCCDA). The microbiological plates were incubated as per manufacturer’s directions. Statistical analyses were calculated in JMP 14.0, with a significance level of *p* ≤ 0.05. Data indicate that all three sources of PAA are effective sanitizers for poultry processing applications compared within treatment. Qualitatively, there were differences in efficacy between the treatments. However, additional studies will be required to determine if those differences are quantitatively distinctive and if they are attributable to differences in product stability.

## 1. Introduction

Food safety is a perpetual challenge for the poultry industry, as carcass contamination is a natural result of processing the live bird into meat. Bacterial foodborne pathogens such as *Salmonella enterica* and *Campylobacter jejuni* do not present clinical signs in broilers, yet can cause gastrointestinal disease in humans [1]. Despite numerous intervention strategies and innovations in place, the foodborne pathogen load within broiler chickens’ microbial load remains largely unpredictable and difficult to control [2]. Therefore, the poultry industry also relies on peri-harvest, during processing, and post-harvest control measures as interventions to safeguard the public against foodborne disease [2].

Microbial reductions during processing require copious amounts of continuously flowing water as well as potent antimicrobials. Antimicrobials used in the poultry processing industry include chlorine, acetic acid, organic acids, and ozone [3]. Often, a multi-hurdle approach is necessary as multiple stages of processing yield hot spots of cross-contamination [4]. The organic acid peracetic acid (PAA) is an important antimicrobial in the poultry industry as it reduces total *Campylobacter* and *Salmonella*, as well as aerobic plate counts (APCs), without extensively altering meat quality [5].

Evisceration is an automated process for poultry processing plants in the United States. Due to feed withdrawal and shipping stress, the viscera are thinned and often ruptured during evisceration. The rupture of the viscera may result in considerable contamination of the carcass by the internal gastrointestinal contents that often harbor foodborne pathogens. Ultimately, the contamination of poultry meat with digesta may be unavoidable [6]. There are multiple stages throughout poultry processing where intervention steps occur and can include spray cabinets that shower the birds as well as immersion tanks. Both systems contain copious amounts of cold water and antimicrobials meant to reduce the microbial load successively throughout processing. Typically, spray interventions are commonly applied immediately prior to the pre-chill or chill system to reduce viscera contamination [6] Additionally, chill tanks are used reduce carcass temperature and often couple the effects of cooling with peracetic acid, which is consistently pumped into the system at a fixed rate in order to reduce microbial contamination during chilling. This is a critical point, as chilling increases the water uptake of the carcass; and, if improperly sanitized, water uptake by the carcass will result in cross-contamination during parts processing [7]. Because PAA degrades quickly in the presence of freshly processed poultry, more stable PAA options may result in better microbial decontamination [8]. Anecdotal evidence suggests that PeraClean (White Paper, Evonik Corporation; Essen, North Rhine-Westphalia, Germany) may have improved stability as compared to its competitors. Therefore, the purpose of this study was to evaluate different PAA products for their ability to reduce microbial and pathogen load (University of Arkansas, Fayetteville, Arkansas, USA). The change in stability was not assessed in this project. By evaluating the total change in APC, coliforms, *Salmonella*, and *Campylobacter*, data will be useable by the poultry industry to determine if the stability of PAA truly impacts food safety [9].

## 2. Materials and Methods

### 2.1. Peracetic Acid Solutions

At the onset of the study, PeraClean (P: 23%; Evonik, Essen, Germany), Competitor 1 (C1: 22%), and Competitor 2 (C2: 22%) peracetic acid solutions were prepared according to manufacturer recommendations. The antimicrobials were prepared at concentrations of 400 or 600 ppm in 200-L tubs and 25 or 45 ppm in 700-L industrial vats. The percentage of PAA per solution was validated by the analytical paperwork that arrived with the PAA, and the calculations were based on the calculated percentage of PAA, not the anticipated percentage. Therefore, the calculation was adjusted for minute differences in the actual percentage of PAA per liter. Titrations were performed to verify the concentrations of the peracetic acid solutions, and recorded results are documented in Table 1 (Peracetic Acid EndPoint ID^®^ Test Kit, Thomas^®^, Swedesboro, NJ, USA).

### 2.2. Processing and Chemical Interventions

**Figure 1:**
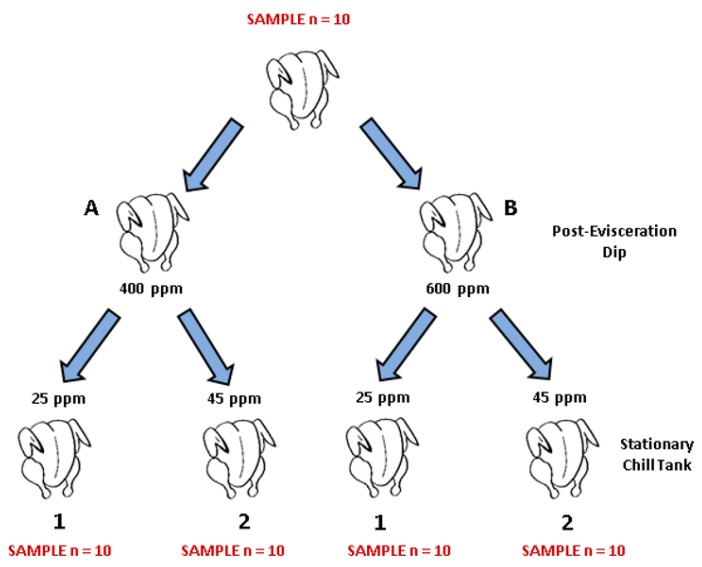
Schematic of the sampling procedure followed in the current experiment. A total of 150 broilers were processed at the University of Arkansas poultry pilot processing plant, where sampling took place. For each PAA sanitizer tested (PeraClean, Competitor 1, and Competitor 2), ten birds were rinsed in 400 mL of neutralizing buffered peptone water (nBPW) for 1 min by manual shaking after manual evisceration took place and were discarded. Following this, forty birds were eviscerated and dipped in solution containing 400 or 600 ppm of PAA for ten seconds immediately after evisceration and then placed into four separate stationary chillers containing either 25 or 45 ppm of PAA sanitizers for 1 h. After birds had been chilled, carcasses were rinsed in nBPW and discarded. Rinsates were collected in specimen cups, stored on ice, and transported to the Center for Food Safety at the University of Arkansas for processing.

A total of 150 Ross 708 birds that were 42 d old were transported to the Pilot Poultry Processing Plant at the University of Arkansas at the onset of the study. According to the corresponding sanitizer (P, C1, C2), 50 birds were processed at a time. Birds were live hung, stunned, manually euthanized by jugular exsanguination, mechanically scalded and de-feathered, and manually eviscerated. Prior to any antimicrobial treatment, 10 birds flushed the line to introduce microbial loads to the processing line, and were not sampled. Additionally, 10 birds were collected prior to processing and after the lines were flushed as the baseline sampling for microbial load for the day. For each antimicrobial treatment group, the first 10 birds were pulled from the line to be sampled immediately following manual evisceration, with water serving as the no-treatment control. The following 10 birds were subjected to either 400 or 600 ppm of the specific sanitizer as a post-evisceration dip for 10 s (A or B, respectively: (Figure 1). Subsequently, the 20 carcasses were divided into to two groups of 10 and either subjected to a stationary chill tank containing 25 or 45 ppm (1 or 2, respectively) of the same sanitizer and chilled for 1 h (Figure 1). The plant was continually in use throughout the experiment and was not disinfected between treatments (P, C1, or C2). While the chiller tanks and post-evisceration tanks were clean, the other components of the facility remained dirty as it was not subjected to additional sanitation steps. Therefore, the use of internal controls was used per treatment for the analysis versus the no-treatment control as a whole. This allowed for consideration of the immediate microbial load entering the system before each sanitation treatment.

### 2.3. Sample Collection

Within each antimicrobial treatment group (groups of 10), carcasses were rinsed in 400 mL of sterile neutralizing buffered peptone water (nBPW) for 1 min by manual agitation (arcing motion) immediately after manually evisceration (C) and following a 1-h chill step in stationary chill tanks containing PAA sanitizers (A1, A2, B1, B2; Figure 1). Carcasses were discarded, rinsates were collected in specimen sample containers, placed on ice, and transported to the Center for Food Safety at the University of Arkansas for downstream analysis.

### 2.4. Microbiological Analysis

Rinsates were stored at 4 °C refrigeration for no more than 18–24 h before microbiological analysis could occur (FSIS Directive 10,250; USDA). All rinsates were diluted ten-fold in 1× phosphate-buffered saline (PBS) in 96-well plates to 10^-7^. Then, 10 µL of diluted rinsates were dot-plated on to xylose lysine deoxycholate agar (XLD), tryptic soy agar (TSA), eosin methylene blue agar (EMB), and modified charcoal cefoperazone deoxycholate agar (mCCDA) and allowed to completely dry before plates were inverted. The XLD, TSA, and EMB plates were incubated for 24 h at 37 °C aerobically and mCCDA plates were incubated for 72 h at 42 °C microaerophilically. Only black colonies on XLD were recorded as *Salmonella*, all colonies on TSA were recorded as the total aerobic bacteria count, both dark purple and green metallic colonies were recorded as total coliforms on EMB, and only silver mucoid colonies were recorded from mCCDA as *Campylobacter*.

### 2.5. Statistical Analysis

Recorded plate counts were both log_10_ transformed and evaluated for the presence or absence of growth on the microbial plates. The effect of treatment location on both log CFU/mL of rinsate, and presence of growth was analyzed in JMP 14.0 using a mixed model (SAS, Cary, NC, USA). Due to the inability to completely clean the processing plant between chemical amendments, each PAA was evaluated separately and within individual treatments. Means were separated using Dunnett’s comparison with a significance value of *p* ≤ 0.05. The effect of treatment location on the presence of growth was analyzed using analysis of means with a significance level of *p* ≤ 0.05. Only statistically significant changes within treatment are presented in this manuscript; the remaining figures are in the Supplemental File.

## 3. Results

Statistical analysis indicated that the microbial load changed throughout processing from the initial baseline microbial load until the end, and that the random effect indicated a bias in the data that made each individual PAA treatment not comparable to the others. Additionally, there were significant differences associated with the actual titrated versus calculated PAA concentrations, which ultimately made direct comparisons difficult to accomplish (Table 1). Together, this information resulted in a much more conservative analysis than initially planned, with the within-treatment comparisons being made directly using statistical methods, and the between-treatment comparisons being qualitative. Fortunately, the microbial load was tracked by treatment, as within-treatment controls were included in the study. Therefore, the necessary data existed to allow us to compare the different PAA treatment concentrations within a specific commercial PAA source. All of the non-significant figures are reported in the Supplemental File (Figures S1–S6). Only significant data are presented in the main manuscript. 

The within-treatment comparison used the samples taken immediately prior to each PAA group treatment, which were set as the control for the microbial load. Each treatment was conducted as per Figure 1, with a post-evisceration dip to simulate the spray cabinet for 10 s, followed by immersion chilling for 1 h.

**Figure 2:**
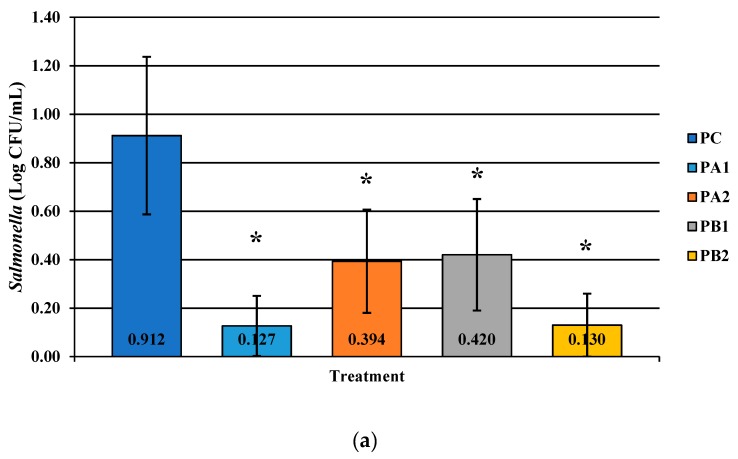

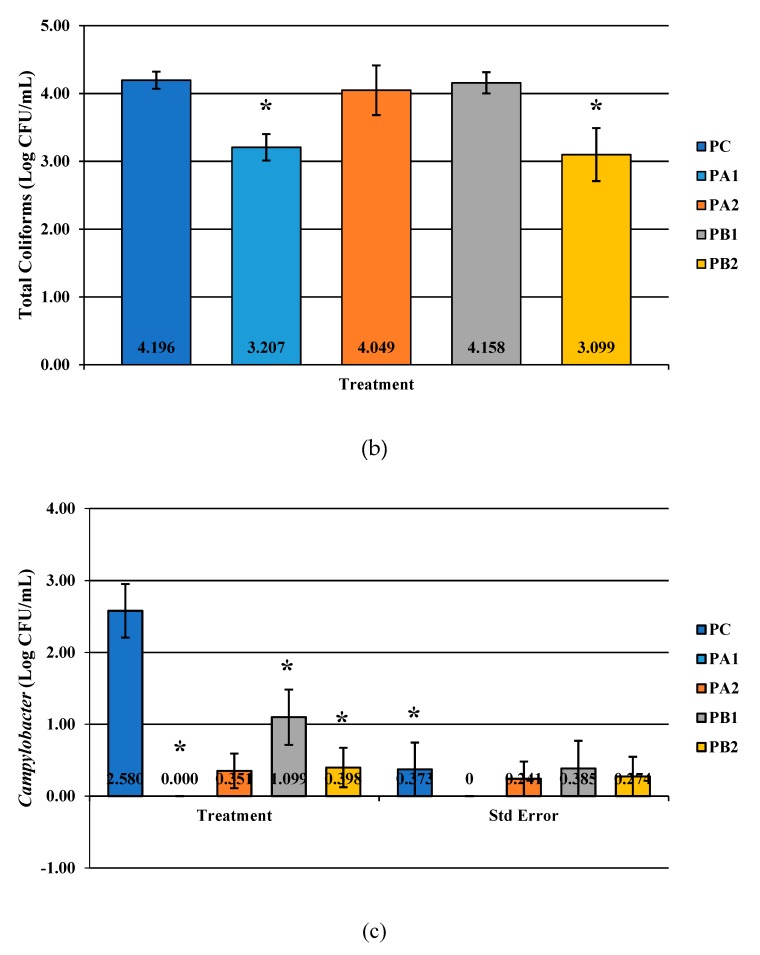
The effect of PeraClean, a PAA acid sanitizer, used as an antimicrobial dip (400 or 600 ppm) and in a stationary chilling tank (25 or 45 ppm) on the load of *Salmonella* (**a**), total coliforms (**b**), and *Campylobacter* (**c**) present in the rinsates of 42-d-old Ross 708 broilers. The means are numerically stated on the bar graph. N = 50, *n* = 10, *p* < 0.0001 (a); N = 50, *n* = 10, *p* < 0.0001 (b); N = 50, *n* = 10, *p* < 0.0001 (c). PA1 refers to a 400 ppm/25 ppm treatment, PA2 refers to a 400 ppm/45 ppm treatment, PB1 refers to a 600 ppm/25 ppm treatment, and PB2 refers to a 600 ppm/45 ppm treatment, with each treatment being first a post-evisceration dip followed by an immersion chill.

**Figure 3:**
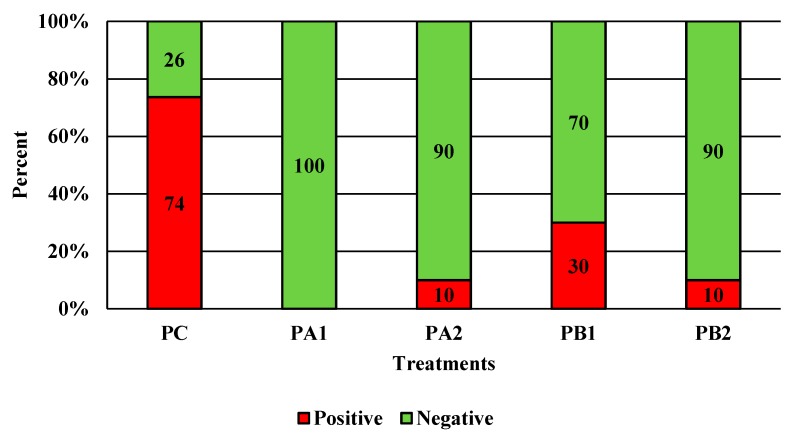
The effect of PeraClean, a PAA sanitizer, used as an antimicrobial dip (400 or 600 ppm) and in a stationary chilling tank (25 or 45 ppm) on the prevalence of *Campylobacter* present in the rinsates of 42-d-old Ross 708 broilers. N = 50, *n* = 10, *p* < 0.0001. PA1 refers to a 400 ppm/25 ppm treatment, PA2 refers to a 400 ppm/45 ppm treatment, PB1 refers to a 600 ppm/25 ppm treatment, and PB2 refers to a 600 ppm/45 ppm treatment, with each treatment being first a post-evisceration dip followed by an immersion chill.

PeraClean exhibited statistically significant reductions in coliforms, *Salmonella*, and *Campylobacter* as compared to the no-treatment control. *Salmonella* was reduced the most from the control by 0.765 log_10_ at PA1, although each PAA treatment group was significantly different from the control as measured by Dunnett’s test (Figure 2A; *p* < 0.0001). Numerically, the coliforms were significantly reduced by 1.097 log_10_ CFU/mL from the original incoming load (Figure 2B; *p* < 0.0001). The PA1 group was able to also completely reduce *Campylobacter* by 2.58 log_10_ CFU/mL of rinsates (Figure 2C; *p* < 0.0001). Interestingly the PA1 group also consistently reduced *Campylobacter* prevalence on spot plates (Figure 3; 100%; *p* < 0.0001), with statistically significant differences observed with each treatment as compared to the initial incoming bacterial load.

**Figure 4:**
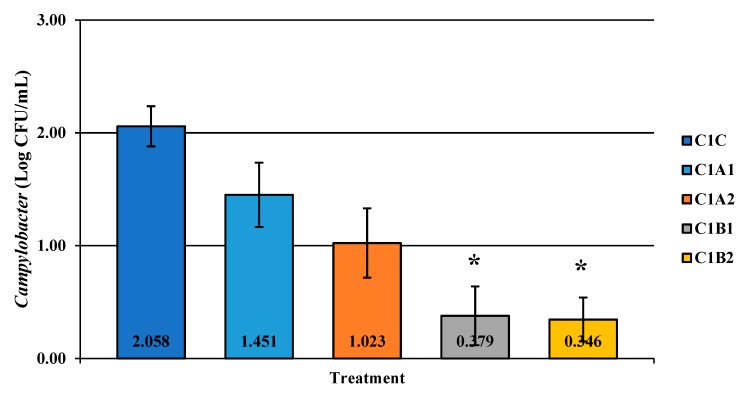
The effect of Competitor 1, a PAA sanitizer used as an antimicrobial dip (400 or 600 ppm) and in a stationary chilling tank (25 or 45 ppm) on the load of *Campylobacter* present in the rinsates of 42-d-old Ross 708 broilers. The means are numerically stated on the bar graph. N = 50, *n* = 10, *p* = 0.0022. CA1 refers to a 400 ppm/25 ppm treatment, CA2 refers to a 400 ppm/45 ppm treatment, CB1 refers to a 600 ppm/25 ppm treatment, and CB2 refers to a 600 ppm/45 ppm treatment, with each treatment being first a post-evisceration dip followed by an immersion chill.

**Figure 5:**
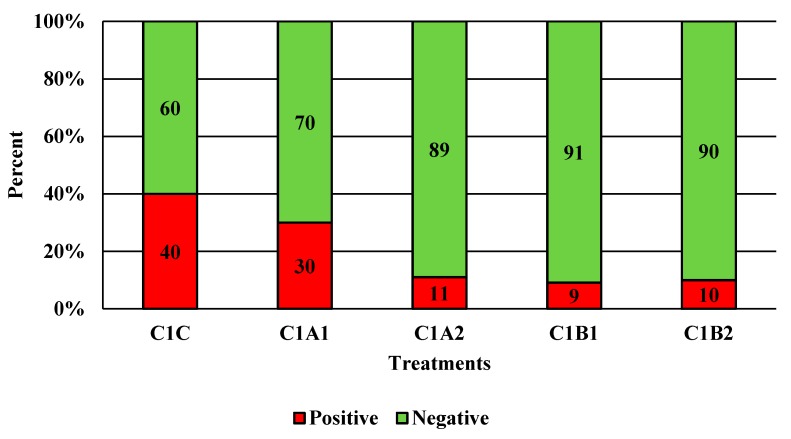
The effect of Competitor 1, a PAA sanitizer used as an antimicrobial dip (400 or 600 ppm) and in a stationary chilling tank (25 or 45 ppm) on the prevalence of *Campylobacter* present in the rinsates of 42-d-old Ross 708 broilers. N = 50, *n* = 10, *p* = 0.0013. CA1 refers to a 400 ppm/25 ppm treatment, CA2 refers to a 400 ppm/45 ppm treatment, CB1 refers to a 600 ppm/25 ppm treatment, and CB2 refers to a 600 ppm/45 ppm treatment, with each treatment being first a post-evisceration dip followed by an immersion chill.

**Figure 6:**
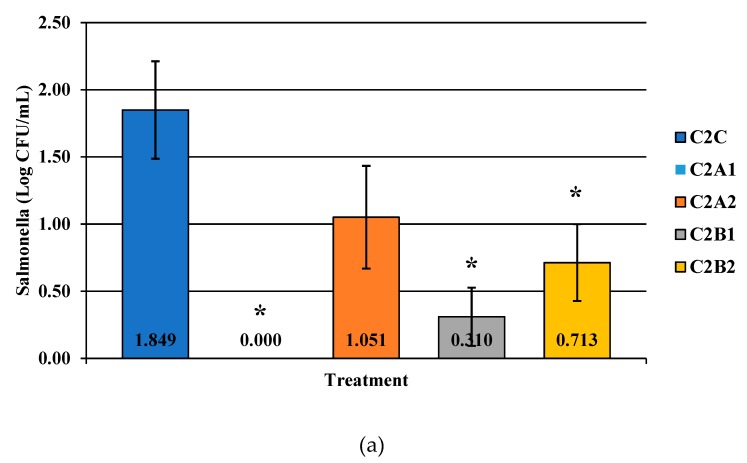

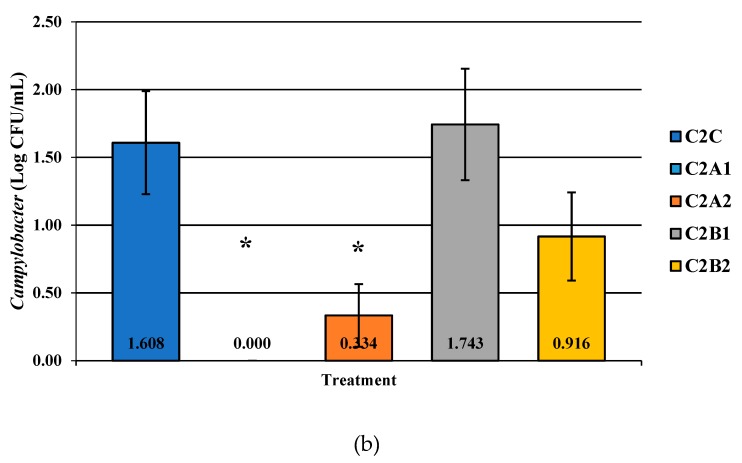
The effect of Competitor 2, a PAA sanitizer used as an antimicrobial dip (400 or 600 ppm) and in a stationary chilling tank (25 or 45 ppm) on the load of *Salmonella* (**a**), total aerobic bacteria (**b**), and *Campylobacter* present in the rinsates of 42-d-old Ross 708 broilers. The means are labeled on the bar graph. The main effect was significant N = 50, *n* = 10, *p* < 0.0001 (a); N = 50, *n* = 10, *p* = 0.0479 (b); N = 50, *n* = 10, *p* = 0.0002 (c). C2A1 refers to a 400 ppm/25 ppm treatment, C2A2 refers to a 400 ppm/45 ppm treatment, C2B1 refers to a 600 ppm/25 ppm treatment, and C2B2 refers to a 600 ppm/45 ppm treatment, with each treatment being first a post-evisceration dip followed by an immersion chill.

**Figure 7:**
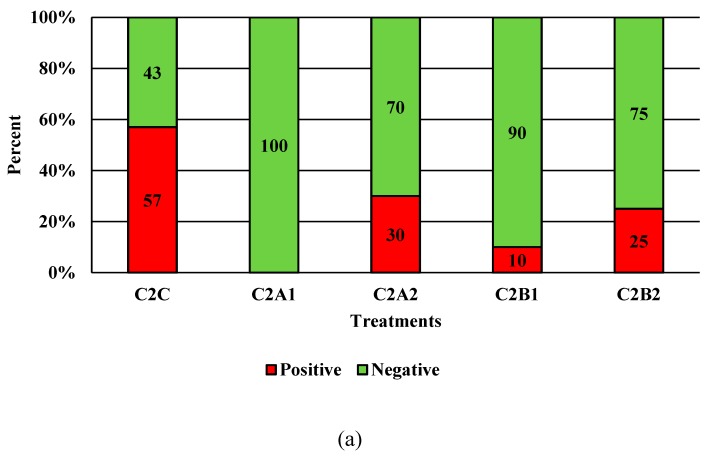

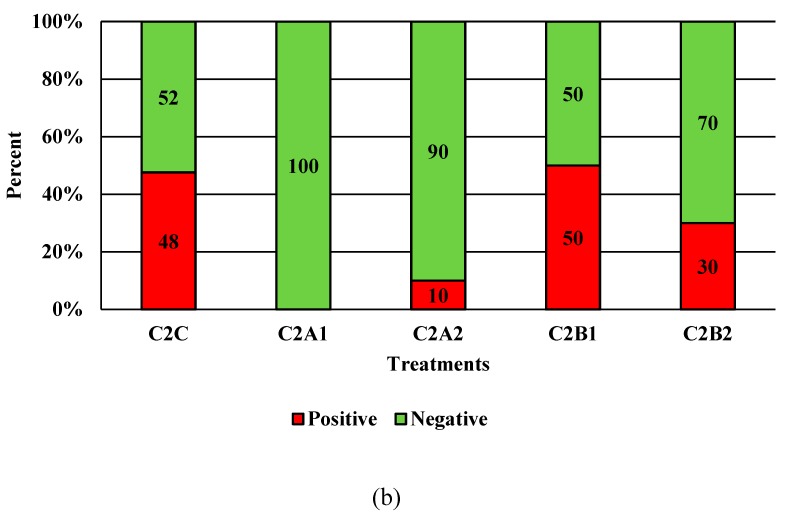
The effect of Competitor 2, a peracetic acid sanitizer used as an antimicrobial dip (400 and 600 ppm) and in a stationary chilling tank (25 and 45 ppm) on the prevalence of *Salmonella* (**a**), and *Campylobacter* (**b**) present in the rinsates of 42 d old Ross 708 broilers. N = 50, *n* = 10, *p* < 0.0001 (**a**); N = 50, *n* = 10, *p* < 0.0001 (**b**).

Competitor 1 exhibited significant reductions with C1B1 and C1B2 for *Campylobacter* alone (Figure 4; *p* < 0.05). Qualitatively, this was less effective than PeraClean, which had significant reductions for *Campylobacter*, *Salmonella*, and coliforms when comparing the original load to the final reduced load. Additionally, the maximum reduction in prevalence was 91% post-treatment for competitor 2 (Figure 5; *p* < 0.05). Competitor 2 had a total reduction in *Campylobacter* in three of the four treatment groups compared to the control, with the greatest reduction of 1.849 log_10_ CFU/mL of rinsate (Figure 6; *p* < 0.001). Prevalence data indicated that C2A1 had the best reduction overall (100%; Figure 7; *p* < 0.001). Unlike PeraClean, prevalence varied from 50% to 100%, and was therefore qualitatively different.

## 4. Discussion

Although the original objective to directly compare PeraClean with the competition was not quantitatively achieved, ultimately in commercial settings PAA titration post-mixing may not be taken into consideration when comparing different PAAs and making management decisions centered around finding the optimal product for the plant. One would assume that the disassociation of the PAA in water to the right concentration (ppm) would be consistent between PAA treatments. However, while the amount of time between chemical mixing and application was relatively consistent for all of the products in this study, the disassociation of the concentrated PAA to its final concentration (ppm) was not consistent. It is possible that many of the within-treatment controls may also have suffered from these effects, which could explain why the lower concentrations were more effective than the higher concentrations. As PAA may have different disassociation rates based on the respective chemistry of each commercial source, the higher doses may actually still not be at their effective range. The proposed effects of stability were somewhat surprising, and remain a point of future discussion. Potentially, the stabilizing products in each of the PAAs from different commercial sources, which are proprietary, may result in the variable effects observed at the plant. Therefore, when considering the changes in microbial load by time as well as the PAA titration points, direct comparisons of sample treatments were not feasible and a qualitative analysis approach was chosen instead.

Importantly, this may be an interesting point for future studies, as calculated titration points and actual titration points at a given time may not be consistent among PAA chemicals from different commercial sources. Therefore, monitoring PAA levels at the plant may not be consistent per PAA, and ultimately the control of PAA levels in a plant may not be as well defined as believed. Additionally, when evaluating products against a specific chemical, stability and time-to-stability are important factors when executing future studies. While unaware of these considerations prior to this study, the analysis of the current study was conducted in an attempt to be fair, balanced, and statistically sound within the confines of the experimental design.

Comparisons between individual treatments (PeraClean, Competitor 1, Competitor 2) were not statistically possible or appropriate due to the difference in the titration versus the calculated point for PAA. Qualitatively, it appears that PeraClean reduced more quantifiable detection groups that the industry monitors, namely, coliforms, *Salmonella*, and *Campylobacter.* The importance of *Salmonella* and *Campylobacter* plate counts are obvious as their reduction ultimately reduces the food safety risk [9] However, coliforms are also used by the industry as a marker of sanitation. Therefore, the reduction of coliforms by PeraClean is extremely important as no other PAA tested on this study was able to accomplish that task, and this potentially indicates that PeraClean is a more effective sanitizing agent. While the reductions in *Campylobacter* and *Salmonella* were also not comparable between groups due to the differences in microbial load entering the system as the day progressed, PeraClean was consistently able to reduce both pathogens. Further research needs to be conducted that enables the direct comparisons of these products with standardized microbial loads and titration points.

While different PAAs are assumed to perform similarly in processing, perhaps the difference in stability of PeraClean ultimately increases its efficacy. Additional studies need to be conducted to evaluate the effects PeraClean has on shelf-life, especially with comparisons to competitor 2, which was able to reduce more pathogens but was unable to significantly change coliform counts. It would be interesting to also include sensory testing to see if the difference in PAA stability impacts meat quality.

## Figures and Tables

**Table 1:** The PAA concentrations of the products as described by the chemistry documentation.

Product	% PAA
PeraClean	23% PAA
Competitor 1	22% PAA
Competitor 2	22% PAA

## Supplementary Materials

The following are available online at https://www.mdpi.com/2076-2607/7/11/503/s1
