# Supplementary material for: The Reduction of Pathogen Load on Ross 708 Broilers when Using Different Sources of Commercial Peracetic Acid Sanitizers in a Pilot Processing Plant
Source: Microorganisms. 2019 Oct 29;7(11):503. doi: 10.3390/microorganisms7110503 (PMC6920778; doi:10.3390/microorganisms7110503)
Supplement: Supplementary file 1 [file microorganisms-07-00503-s001.zip › microorganisms-584472-SI.pdf]

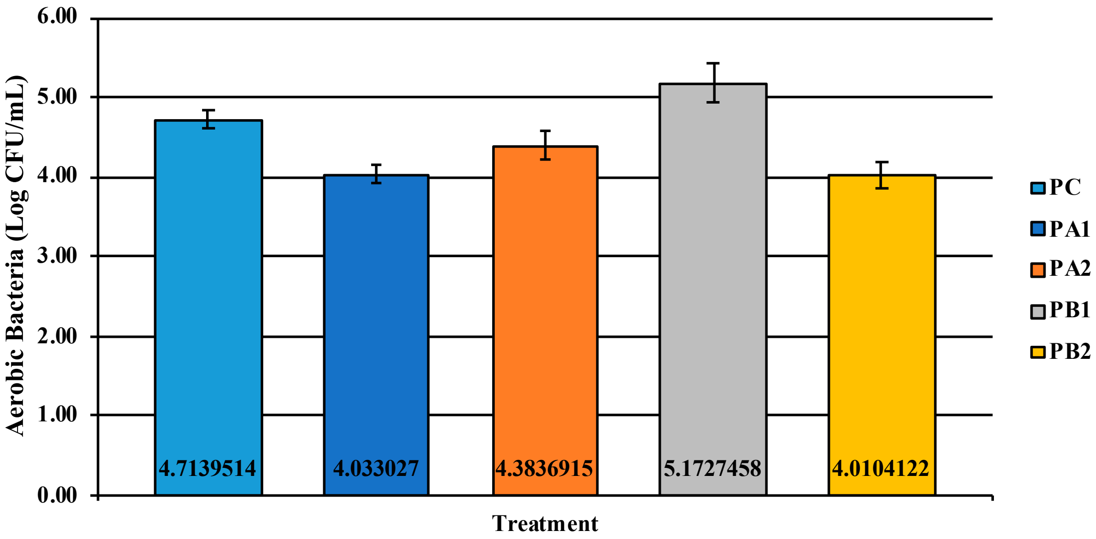

Figure 1: The effect of Peraclean, a peracetic acid sanitizer, used as an antimicrobial dip (400 and 600 ppm) and in a stationary chilling tank (25 and 45 ppm) on the load of aerobic bacteria present in the rinsates of 42 d old Ross 708 broilers. N = 50, n = 10, P > 0.05.

A

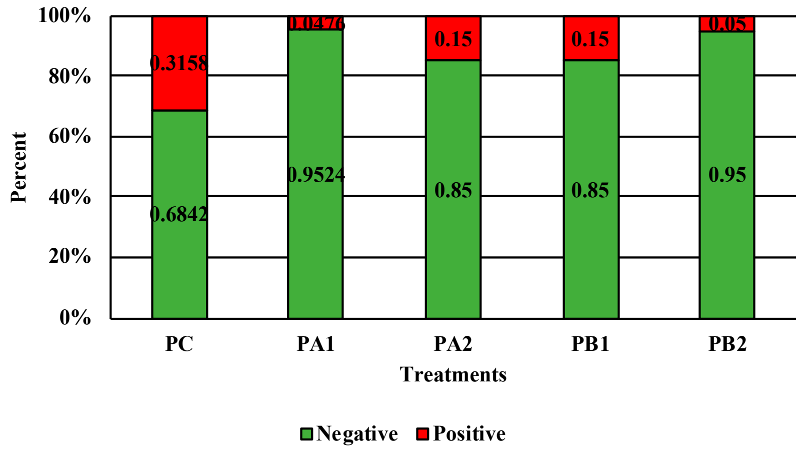

B

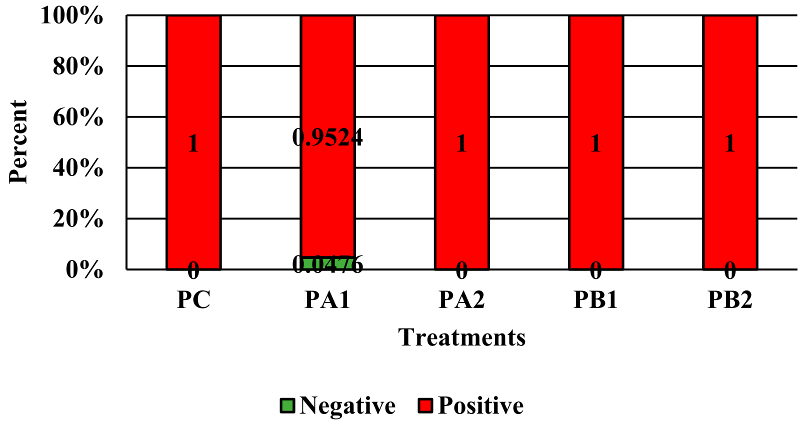

C

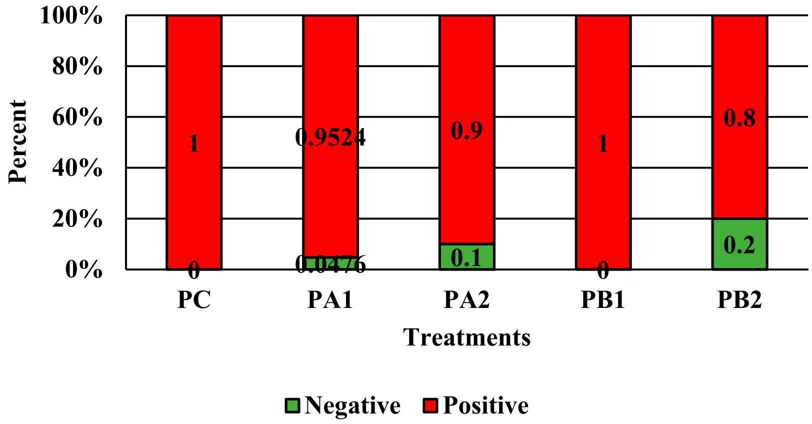

Supplemental Figure 2:
The effect of Peraclean, a peracetic acid sanitizer, used as an antimicrobial dip (400 and 600 ppm) and in a stationary chilling tank (25 and 45 ppm) on the prevalence of *Salmonella* (a), aerobic bacteria (b), and total coliforms (c) present in the rinsates of 42 d old Ross 708 broilers. (a) N = 50, n = 10, P > 0.05; (b) N = 50, n = 10, P > 0.05; (c) N = 50, n = 10, P > 0.05.

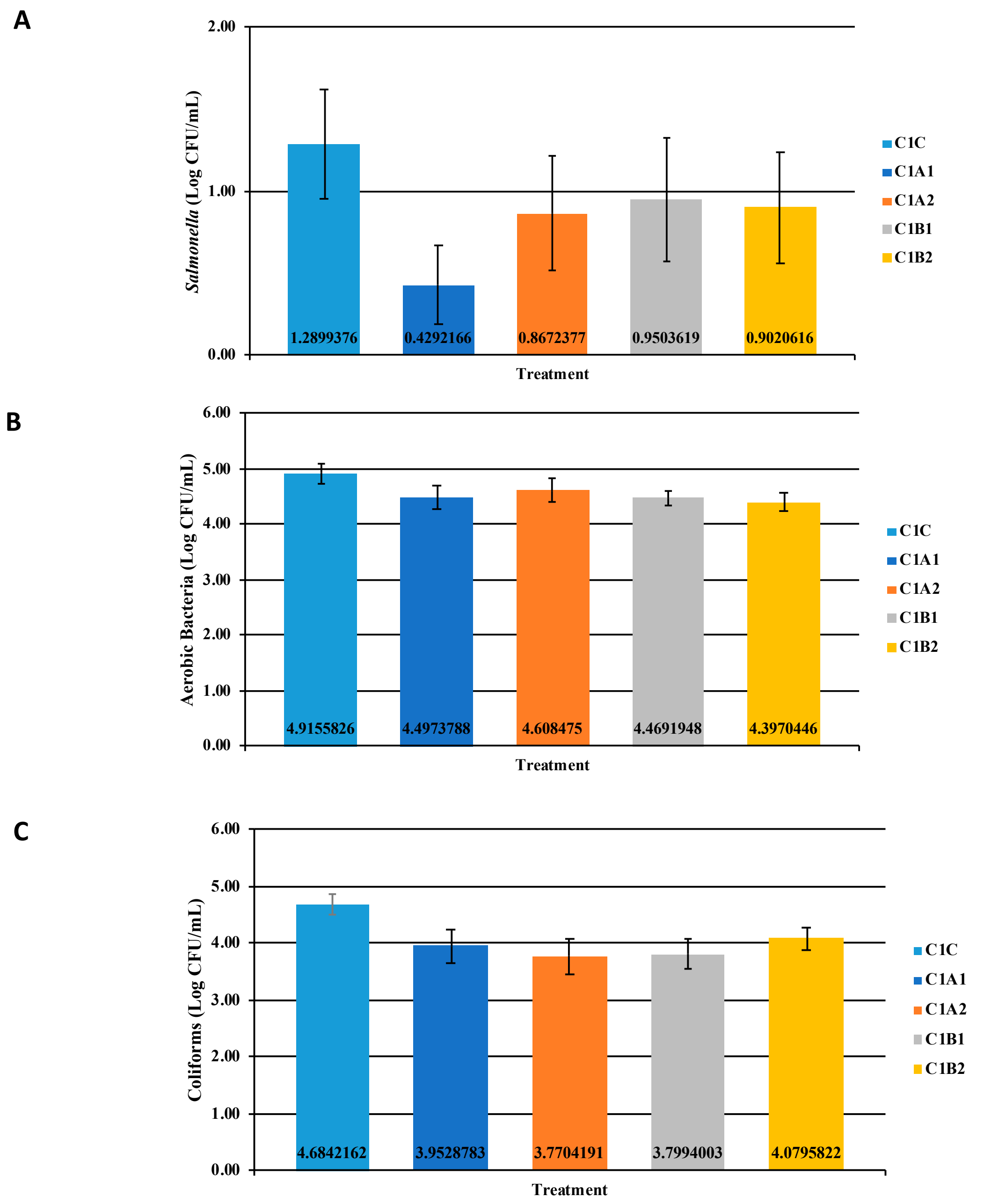

Supplemental Figure 3:

The effect of Competitor 1, a peracetic acid sanitizer used as an antimicrobial dip (400 and 600 ppm) and in a stationary chilling tank (25 and 45 ppm) on the load of *Salmonella* (a), aerobic bacteria (b), and total coliforms (c) present in the rinsates of 42 d old Ross 708 broilers. (a) N = 50, n = 10, $P > 0.05$; (b) N = 50, n = 10, $P > 0.05$; (c) N = 50, n = 10, $P > 0.05$.

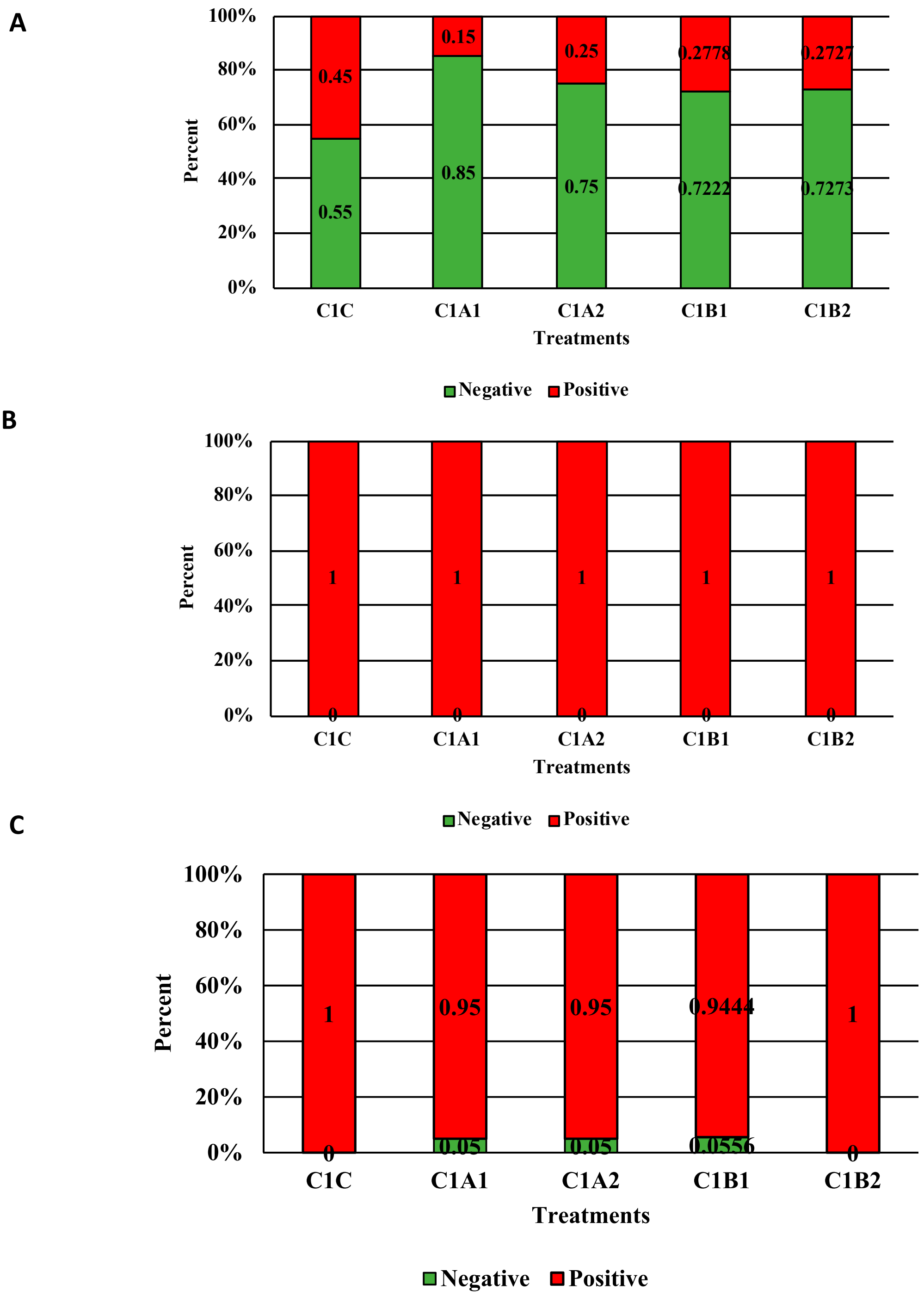

Supplemental Figure 4:
The effect of Competitor 1, a peracetic acid sanitizer used as an antimicrobial dip (400 and 600 ppm) and in a stationary chilling tank (25 and 45 ppm) on the prevalence of Salmonella (a), aerobic bacteria (b), and total coliforms (c) present in the rinsates of 42 d old Ross 708 broilers. (a) N = 50, n = 10, P > 0.05; (b) N = 50, n = 10, P > 0.05; (c) N = 50, n = 10, P > 0.05.

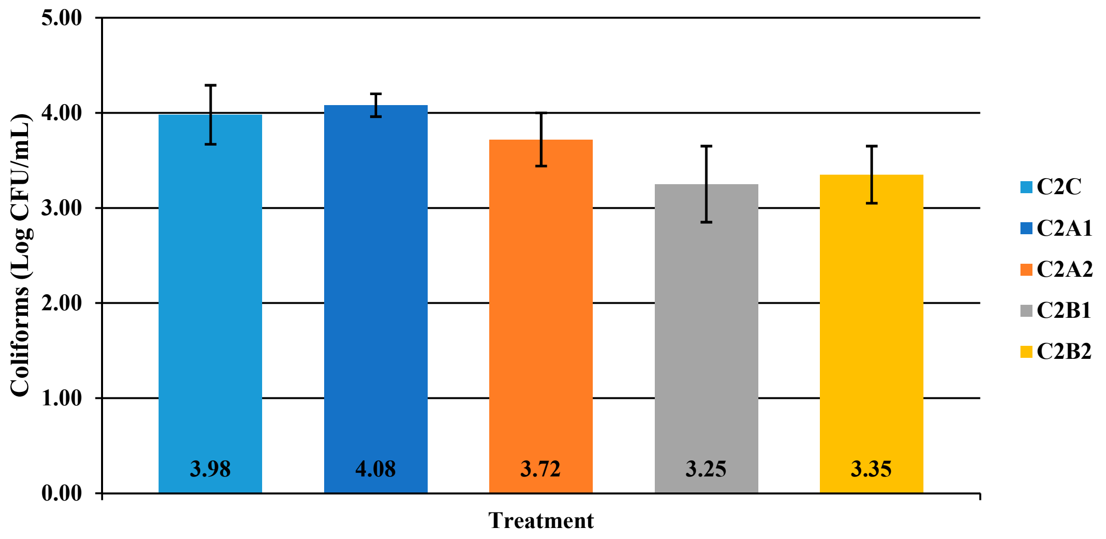

Supplemental Figure 5:
The effect of Competitor 2, a peracetic acid sanitizer used as an antimicrobial dip (400 and 600 ppm) and in a stationary chilling tank (25 and 45 ppm) on the load of *total coliforms* present in the rinsates of 42 d old Ross 708 broilers. N = 50, n = 10, P > 0.05.

A

B

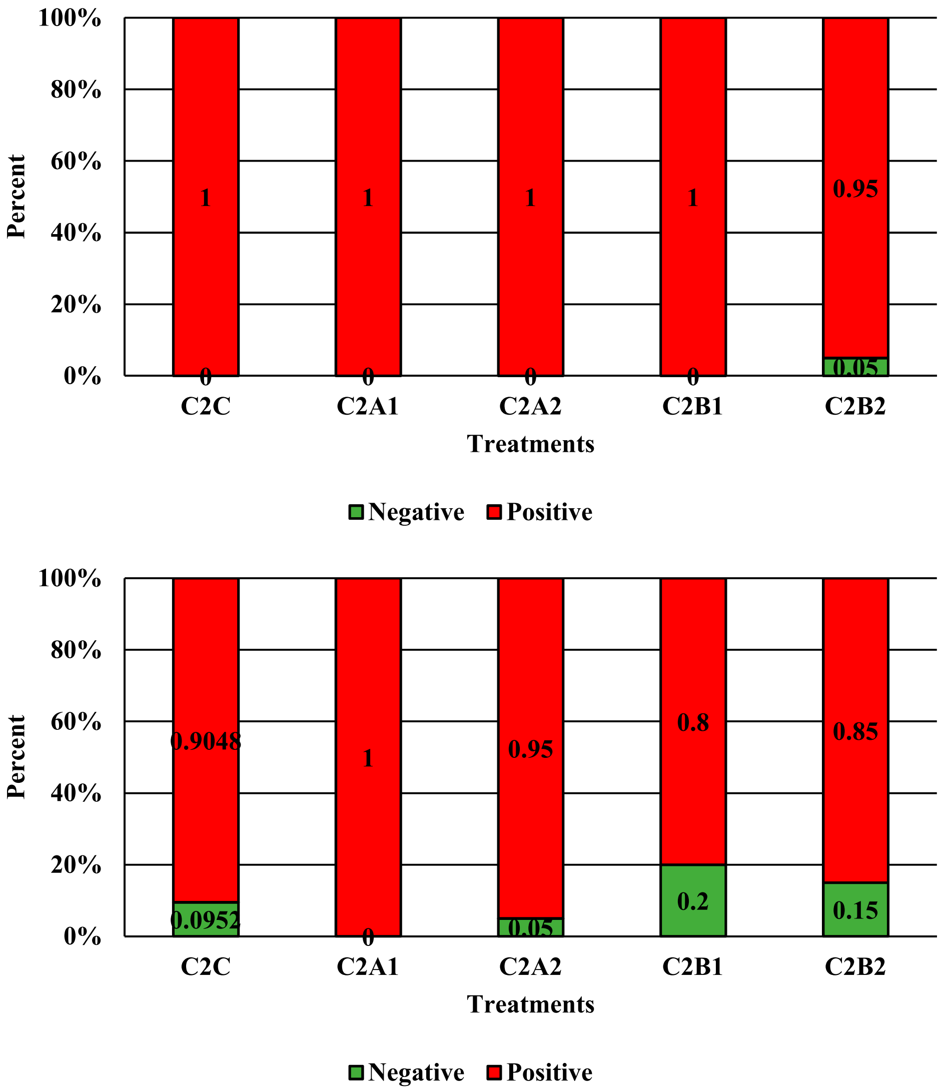

Supplemental Figure 6:

The effect of Competitor 2, a peracetic acid sanitizer used as an antimicrobial dip (400 and 600 ppm) and in a stationary chilling tank (25 and 45 ppm) on the prevalence of aerobic bacteria (a), and total coliforms (b) present in the rinsates of 42 d old Ross 708 broilers. N = 50, n = 10, P > 0.05 (a); N = 50, n = 10, P > 0.05 (b).
